# AmiA and AliA peptide ligands, found in *Klebsiella pneumoniae*, are imported into pneumococci and alter the transcriptome

**DOI:** 10.1038/s41598-024-63217-2

**Published:** 2024-05-30

**Authors:** Janine Lux, Lucía Sánchez García, Patricia Chaparro Fernández, Laura Laloli, Manon F. Licheri, Clement Gallay, Peter W. M. Hermans, Nicholas J. Croucher, Jan-Willem Veening, Ronald Dijkman, Daniel Straume, Lucy J. Hathaway

**Affiliations:** 1 Institute for Infectious Diseases, Faculty of Medicine, University of Bern, Friedbühlstrasse 25, CH-3001 Bern, Switzerland; 2 Graduate School for Cellular and Biomedical Sciences, University of Bern, Bern, Switzerland; 3 Department of Fundamental Microbiology, University of Lausanne, Lausanne, Switzerland; 4 Julius Centre for Health Sciences and Primary Care, University Medical Centre Utrecht (UMCU), Utrecht, The Netherlands; 5 MRC Centre for Global Infectious Disease Analysis, White City Campus, Imperial College London, Sir Michael Uren Hub, London, UK; 6 Multidisciplinary Center for Infectious Diseases, University of Bern, Bern, Switzerland; 7 Microscopy Imaging Centre (MIC), Theodor Kocher Institute, University of Bern, Bern, Switzerland; 8 European Virus Bioinformatics Center, Jena, Germany; 9 Faculty of Chemistry, Biotechnology and Food Science, Norwegian University of Life Sciences, 1430 Ås, Norway

**Keywords:** Cell biology, Microbiology

## Abstract

*Klebsiella pneumoniae* releases the peptides AKTIKITQTR and FNEMQPIVDRQ, which bind the pneumococcal proteins AmiA and AliA respectively, two substrate-binding proteins of the ABC transporter Ami-AliA/AliB oligopeptide permease. Exposure to these peptides alters pneumococcal phenotypes such as growth. Using a mutant in which a permease domain of the transporter was disrupted, by growth analysis and epifluorescence microscopy, we confirmed peptide uptake via the Ami permease and intracellular location in the pneumococcus. By RNA-sequencing we found that the peptides modulated expression of genes involved in metabolism, as pathways affected were mostly associated with energy or synthesis and transport of amino acids. Both peptides downregulated expression of genes involved in branched-chain amino acid metabolism and the Ami permease; and upregulated fatty acid biosynthesis genes but differed in their regulation of genes involved in purine and pyrimidine biosynthesis. The transcriptomic changes are consistent with growth suppression by peptide treatment. The peptides inhibited growth of pneumococcal isolates of serotypes 3, 8, 9N, 12F and 19A, currently prevalent in Switzerland, and caused no detectable toxic effect to primary human airway epithelial cells. We conclude that pneumococci take up *K. pneumoniae* peptides from the environment via binding and transport through the Ami permease. This changes gene expression resulting in altered phenotypes, particularly reduced growth.

## Introduction

*Streptococcus pneumoniae* (pneumococcus) is a Gram positive bacterium listed amongst the priority pathogens by the World Health Organization in 2017^1^. It is the most common cause of bacterial community-acquired pneumonia globally and causes meningitis and septicaemia with significant morbidity and mortality particularly in young children, the elderly and the immunocompromised ^2^. Current vaccines only protect from a fraction of pneumococcal serotypes and not from non-vaccine serotypes or nonencapsulated pneumococci with antibiotic resistance becoming a growing concern^3^.

It has been proposed that *S. pneumoniae* senses the environment via its ABC transporter Ami-AliA/AliB permease by uptake of oligopeptides, modulating gene expression^4^ and nasopharyngeal colonization^5^. By expressing and purifying the transporter's oligopeptide binding proteins AmiA and AliA as recombinant proteins, we identified the peptides AKTIKITQTR and FNEMQPIVDRQ as their respective ligands from nasal wash of children^6^. The peptide sequences matched ribosomal proteins of *Klebsiella pneumoniae* and were found in the secretome of *K. pneumoniae*^7^. The peptides altered pneumococcal phenotypes as they reduced pneumococcal growth, capsule size and transformation rate^8^. Mutation of the genes encoding the oligopeptide binding proteins in strain D39 abrogated the peptide effect on pneumococcal growth^7,8^, therefore peptide interaction with the oligopeptide binding proteins of the Ami permease is essential for the effect. This is in line with the identification of pneumococcal strains which have mutations in the oligopeptide-binding proteins of the Ami permease and whose growth is not inhibited by the peptides, such as the previously-identified R6 and ATCC 700903^7^.

Here, we aimed to determine whether uptake of the AmiA and AliA peptides via the Ami-AliA/AliB permease is necessary for their effect and to find clues about their mode of action by exploring changes at the transcriptional level. By using fluorescently labelled peptides, we show that the peptides are taken up into the pneumococci and this uptake was necessary to suppress bacterial growth. Both peptides altered the pneumococcal transcriptome with similar but distinct effects.

## Results

### Pneumococcal growth inhibition by AmiA and AliA peptide ligands requires uptake via Ami-AliA/AliB permease

**Figure 1:**
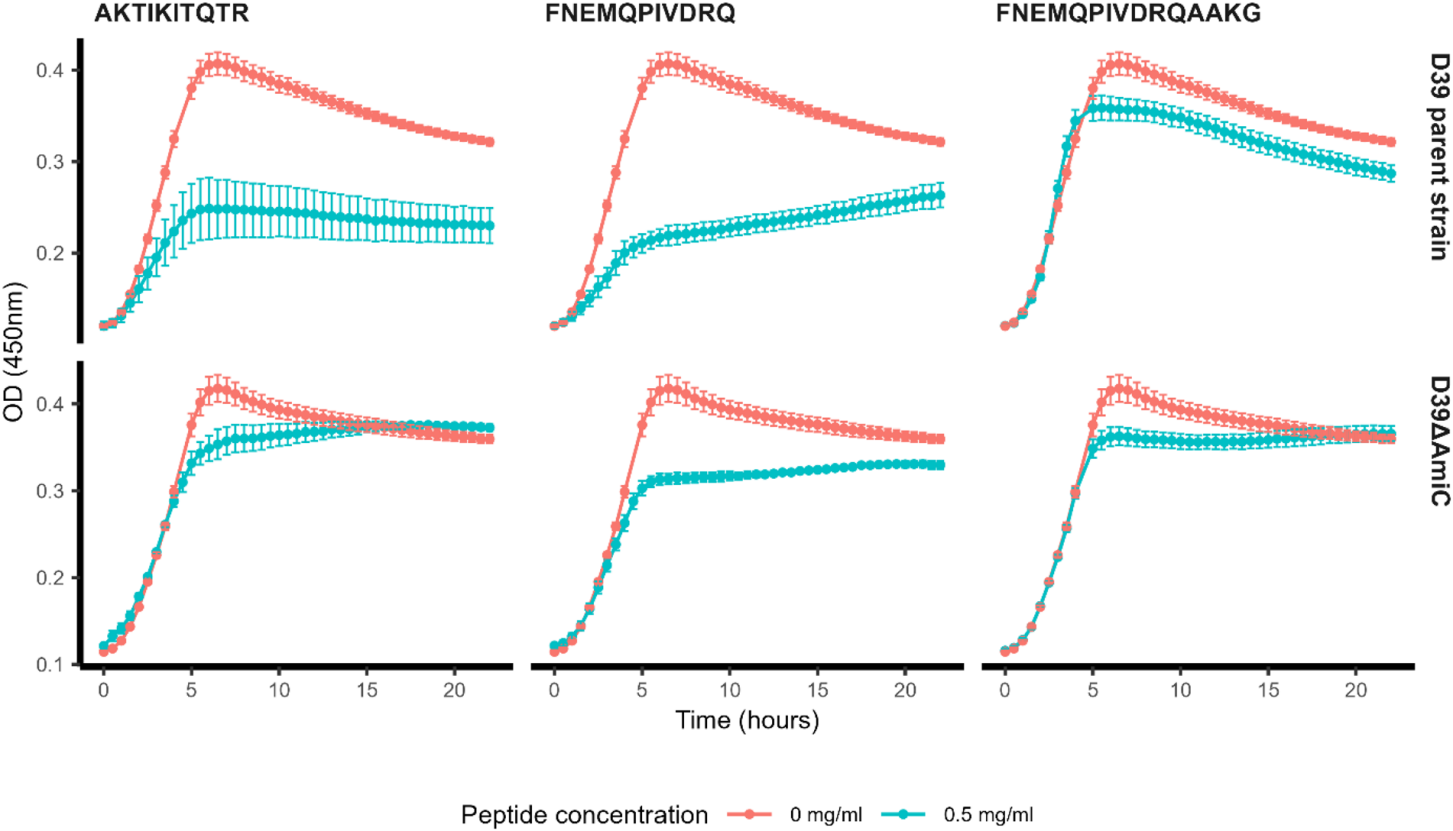
Growth suppression requires uptake of AmiA and AliA ligands via the Ami permease. The *S. pneumoniae* D39 parent strain was inhibited by AmiA ligand (AKTIKITQTR) and AliA ligand (FNEMQPIVDRQ), but inhibition was greatly reduced in the ∆*amiC* mutant. This indicates that the peptides are taken up via the Ami permease to act. Nonspecific ligand control, FNEMQPIVDRQAAKG, had little effect on growth of D39 parent strain or the ∆*amiC* mutant. Growth curves were performed in peptide-free chemically defined medium (CDM) by measuring optical density OD (450 nm) over time in absence or presence of peptide (0.5 mg/ml). Results represent 3 independent experiments, error bars indicate SEM.

**Figure 2:**
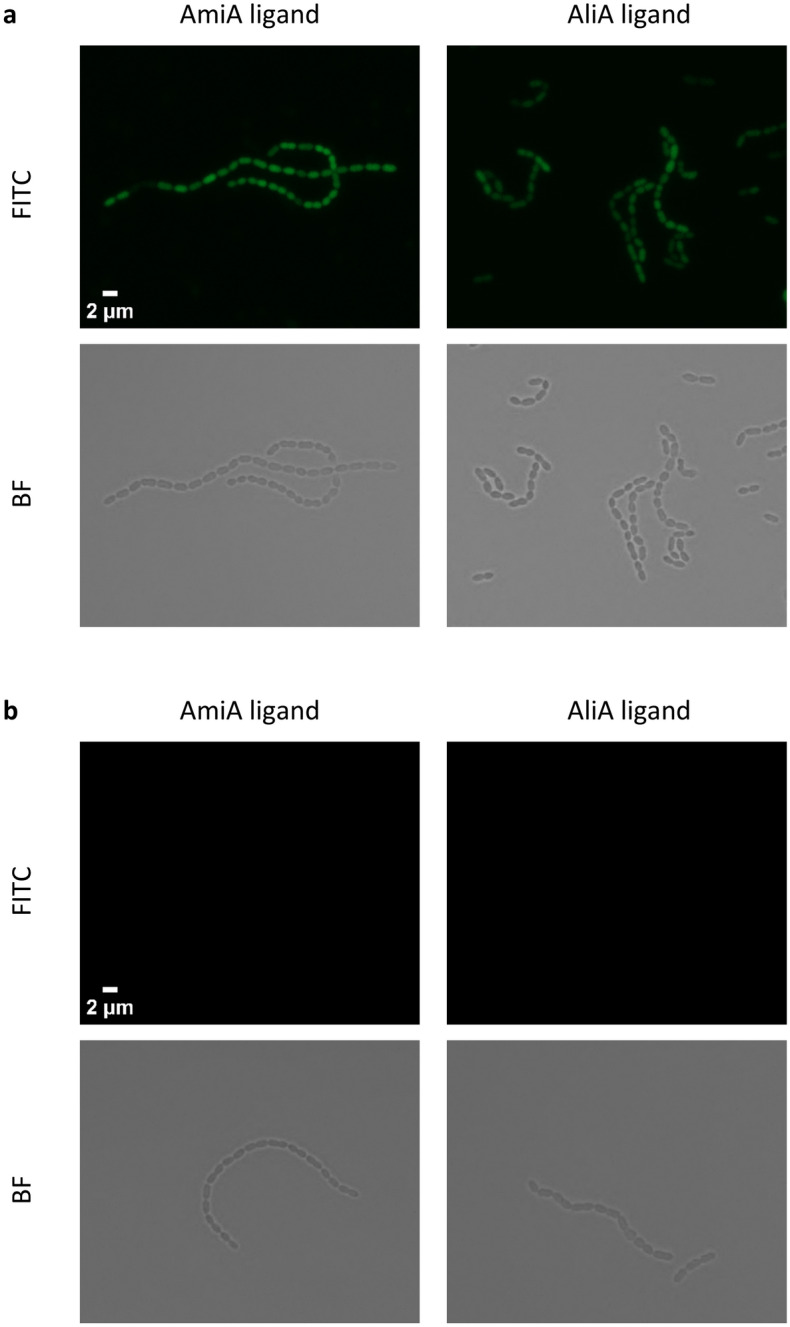
AmiA and AliA peptide ligands are taken up via Ami permease. Peptides located inside of *S. pneumoniae* D39 parent strain **(a)**, but not ∆*amiC* mutant **(b).** Representative images of pneumococcal cells after incubation with FITC-labelled AmiA and AliA peptide ligands taken at mid-bacterium localization in z position showing FITC and Brightfield (BF) channel. Scale bar indicates 2 µm for all pictures in a and b.

**Figure 3:**
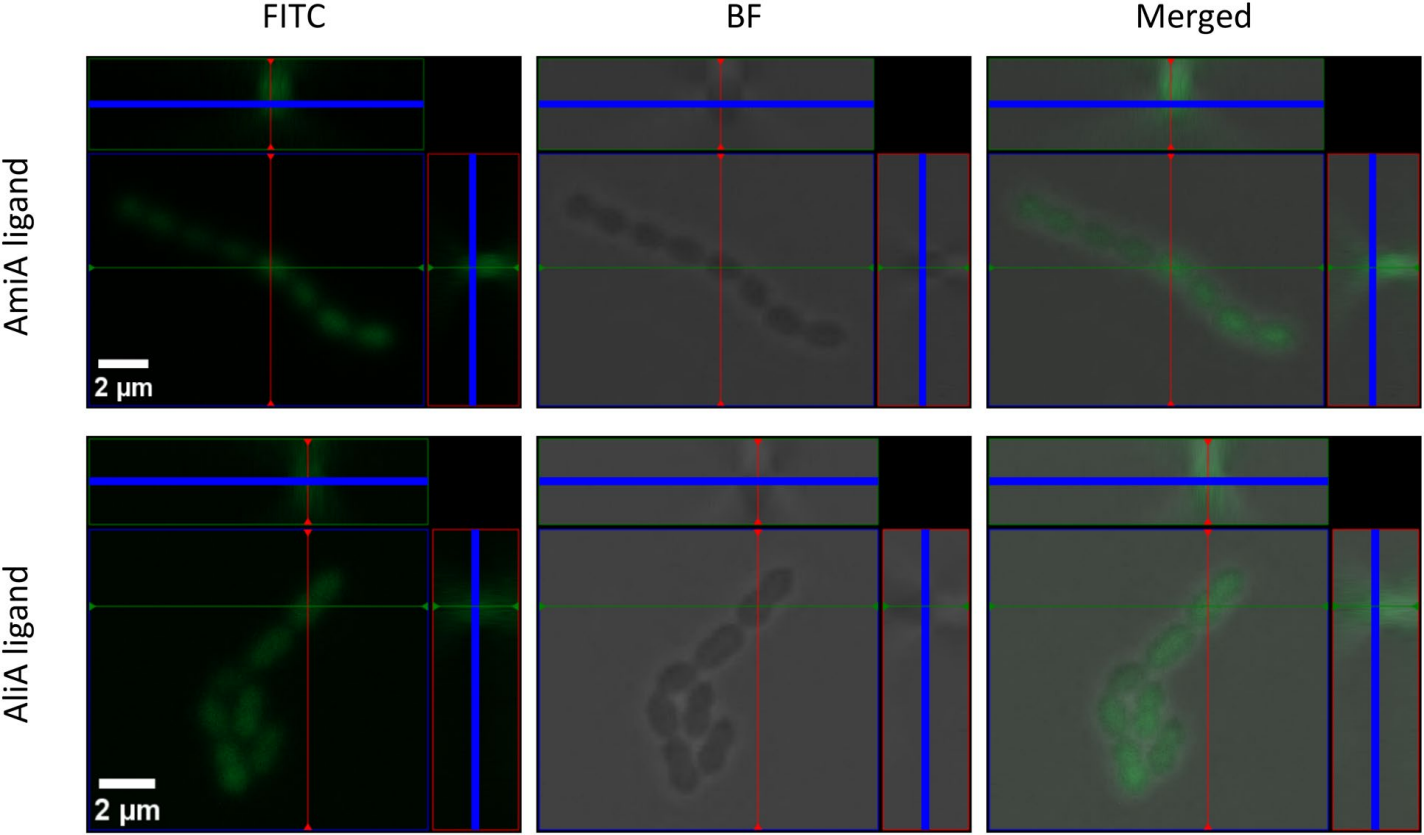
AmiA and AliA peptide ligands locate inside pneumococcal cells of strain D39. Representative images of *S. pneumoniae* strain D39 parental strain after 5 min incubation with FITC-labelled AmiA and AliA peptide ligands. Scale bar indicates 2 µm for all pictures treated with the same peptide. Orthogonal view from z stack shows colocalization of peptide in FITC channel with cytosol of bacterium in Brightfield (BF) channel.

As the role of the Ami permease is to import oligopeptides, we hypothesized that uptake may be necessary for response to the peptides. We confirmed this using a mutant in which one of the two permease subunits, AmiC, was disrupted. The AmiA and AliA peptide ligands inhibited the *S. pneumoniae* D39 parent strain, but growth suppression was lost or greatly reduced in the Δ*amiC* mutant (Fig. 1). A nonspecific ligand control had little effect on growth of either parent strain or its AmiC mutant (Fig. 1). The AmiC mutant had no growth deficiency compared to its parent strain in the absence of peptide (Supplementary Fig. S1). To confirm peptide uptake by *S. pneumoniae* and that this requires a functional Ami-AliA/AliB oligopeptide permease, we incubated the D39 parent strain and Δ*amiC* mutant for 5 min with FITC-labelled peptides and performed epifluorescence microscopy. In the D39 strain with a functional Ami permease, but not in the Δ*amiC* mutant, we found FITC staining intracellularly (Fig. 2) confirming peptide uptake. In the orthogonal view of a z-stack, the FITC peptide staining was inside the bacterial cell and not on the membrane (Fig. 3). We did not find FITC staining intracellularly in a D39 Δ*amiA* mutant strain (Supplementary Fig. S2). Thus, peptide uptake via a functional Ami permease is required for pneumococcal growth suppression by the AmiA and AliA peptide ligands.

### AmiA and AliA peptide ligands affect pneumococcal transcriptome

**Figure 4:**
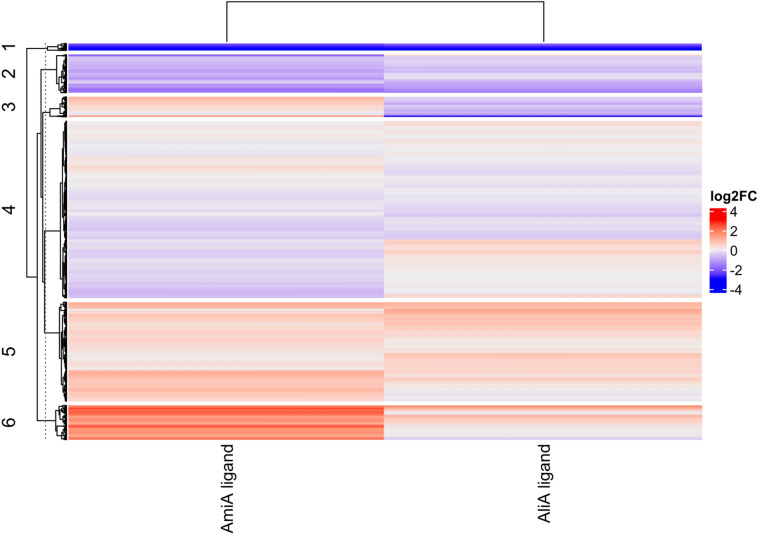
AmiA and AliA peptide ligands cause differential gene expression in *S. pneumoniae* strain D39. AmiA and AliA peptide ligands caused similar changes (upregulation or downregulation) in clusters 1,2,5 and 6, whereas AmiA peptide ligand upregulated and AliA peptide ligand downregulated more genes in cluster 3. Patterns are also different in regions of cluster 4.

To identify the underlying mechanisms by which the AmiA and AliA peptide ligands caused phenotypic changes such as growth inhibition and decreased capsule size and transformation rate^8^, we identified differentially expressed genes in *S. pneumoniae* strain D39 treated with AmiA or AliA peptide ligands compared to the untreated control. AmiA ligand caused more dramatic gene expression changes than AliA ligand as shown in the heatmap (Fig. 4). There, we clustered the differentially expressed genes and identified biological process pathway enrichment within the clusters. Both peptides strongly downregulated genes in cluster 1, which is enriched for peptide and protein transport, branched-chain amino acid (BCAA) biosynthesis and amino acid biosynthesis. Cluster 2, enriched for aromatic amino acid biosynthesis, tryptophan biosynthesis, glycolysis, amino acid biosynthesis and stress response, was downregulated by both peptides, with stronger log2FC (log2 fold change) values for the AmiA ligand. Cluster 3 was not enriched for any biological process, but for the molecular function ribonucleoprotein, ribosomal protein, rRNA binding, RNA binding and upregulated by the AmiA ligand, but downregulated by the AliA ligand. Cluster 4, enriched for cell wall biogenesis/degradation, cell shape, DNA repair, DNA damage, rRNA processing and protein biosynthesis, contained overall more downregulated genes and did not show the same pattern for both peptide treatments. Cluster 5, enriched for transport, sugar transport and phosphotransferase system, included more upregulated genes for both peptide treatments. Cluster 6, enriched for purine and pyrimidine biosynthesis, was strongly upregulated by the AmiA ligand, but not by the AliA ligand.

To better understand which biological processes are represented by the top differentially expressed genes by each peptide treatment, we identified enrichment in the datasets of significantly (p value < 0.05) downregulated (log2FC ≤ -1.25) genes and separately significantly upregulated (log2FC ≥ 1.25) genes (Supplementary Fig. S3). Among the top upregulated genes by both peptides were those involved in the fatty acid biosynthesis process and malonyl-CoA biosynthesis. Expression of genes was increased in additional processes by AmiA ligand, including inosine monophosphate (IMP), uridine monophosphate (UMP), purine and pyrimidine biosynthesis. The process enrichment of the top downregulated genes reflects findings from the heatmap clusters including amino acid synthesis processes for isoleucine and valine, which are both BCAAs, enriched in the dataset of both peptide treatments. Furthermore, threonine biosynthesis, peptide and protein transport processes were enriched in the top downregulated genes by both peptide treatments.

**Figure 5:**
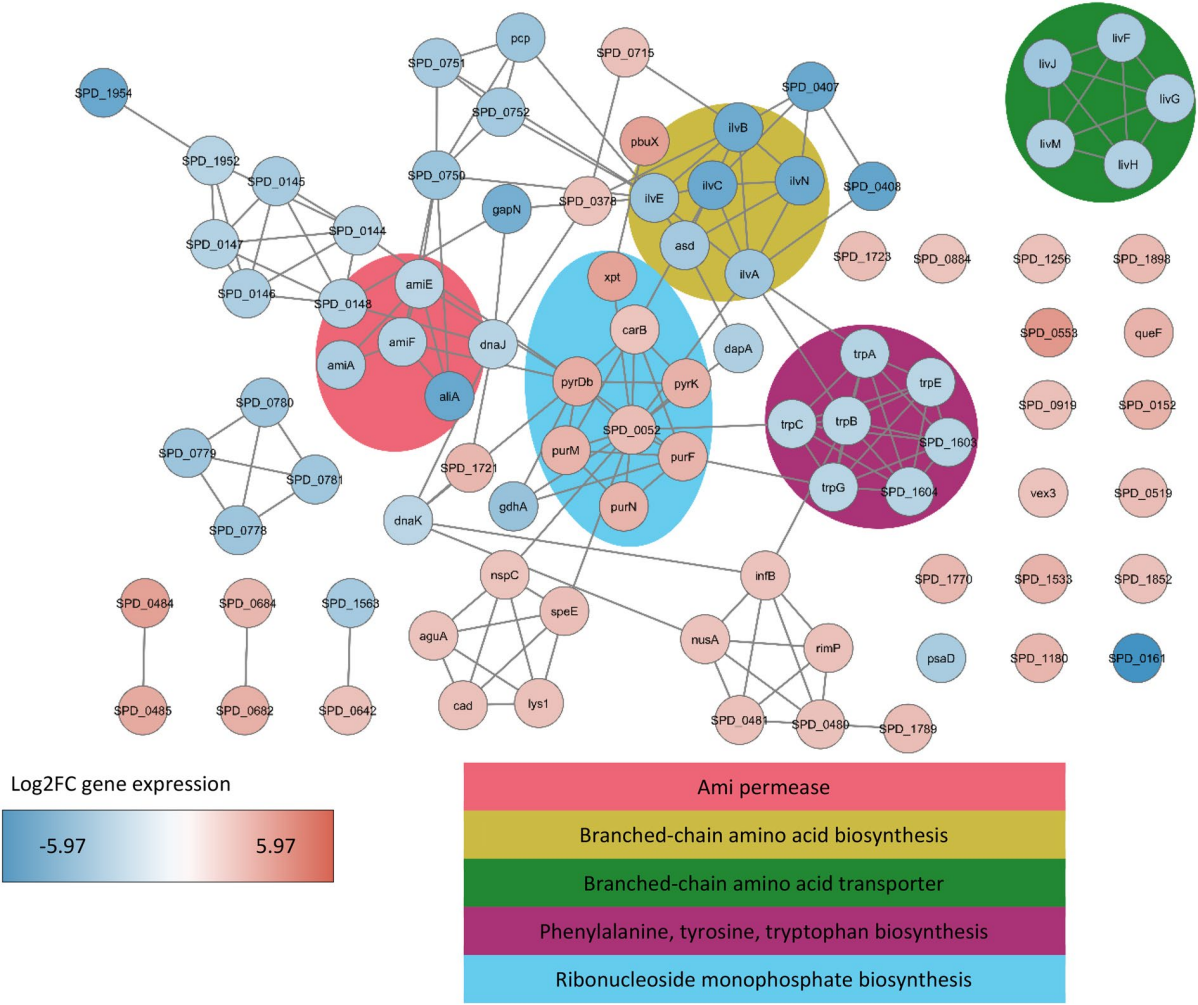
Top downregulated and upregulated genes by AmiA peptide ligand. Gene association network of significantly (p value < 0.05) downregulated (log2FC ≤ -2) and upregulated (log2FC ≥ 2) genes in *S. pneumoniae* strain D39 after 15 min of incubation with AmiA peptide ligand. Lines (edges) represent interactions between 2 genes (nodes) and were imported from the STRING database. Nodes are colour coded according to log2FC expression change: blue represents negative log2FC (downregulated) and red positive log2FC (upregulated). Gene Ontologies or keywords of groups of genes are highlighted in the background.

**Figure 6:**
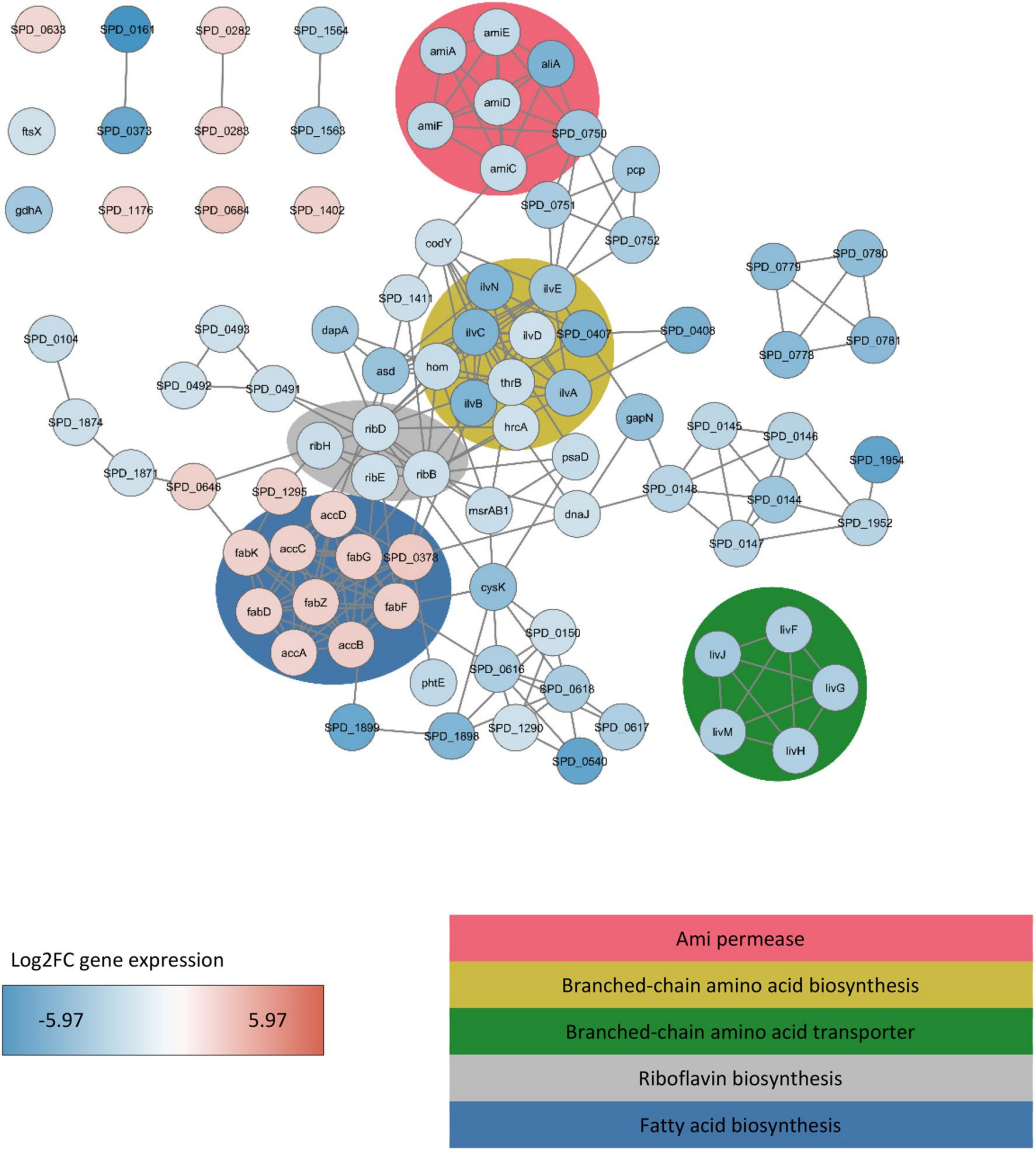
Top downregulated and upregulated genes by AliA peptide ligand. Gene association network of significantly (p value < 0.05) downregulated (log2FC ≤ -1.25) and upregulated (log2FC ≥ 1.25) genes in *S. pneumoniae* strain D39 after 15 min of incubation with AliA peptide ligand. Lines (edges) represent interactions between 2 genes (nodes) and were imported from the STRING database. Nodes are colour coded according to log2FC expression change: blue represents negative log2FC (downregulated) and red positive log2FC (upregulated). Gene Ontologies or keywords of groups of genes are highlighted in the background.

We made a gene interaction network with the top differentially expressed genes, for AmiA ligand in Fig. 5 (− 2 ≥log2FC≥ 2) and for AliA ligand in Fig. 6 (− 1.25 ≥log2FC≥ 1.25). Among genes downregulated by both peptides we see the following clusters: Ami permease (*ami* genes and *aliA*), BCAA biosynthesis (*ilv* genes) and BCAA transporter (*liv* genes), by AmiA ligand in addition phenylalanine, tyrosine, tryptophan biosynthesis (*trp* genes) and by AliA ligand riboflavin biosynthesis (*rib* genes). Among genes upregulated by AmiA ligand we see ribonucleoside monophosphate biosynthesis (*pur* and *pyr* genes) and by AliA ligand fatty acid biosynthesis (*fab* genes). We confirmed significant downregulation of *aliA*, *ilvB* and *livJ* and significant upregulation of *fabK* by real-time RT-PCR for both peptide treatments (Supplementary Fig. S4).

On the single gene level, the top downregulated gene was SPD_0161 (NRAMP family Mn2+/Fe2+ transporter) by both peptides with < -5 log2FC. The top upregulated gene was SPD_0553 (hypothetical protein) by AmiA ligand (3.76 log2FC) and SPD_0684 (bioY, Substrate-specific component BioY of biotin ECF transporter) by AliA ligand (1.88 log2FC). We did not find any *com* genes differentially expressed, but found several single genes significantly downregulated by both peptides with log2FC values in Supplementary Table S1. In summary, the AmiA and AliA peptide ligands altered the pneumococcal transcriptome, affecting transcriptional regulators and downregulating genes for amino acid and protein metabolism.

### AmiA and AliA peptide ligands inhibited growth of clinical isolates of currently prevalent serotypes of *S. pneumoniae*

**Figure 7:**
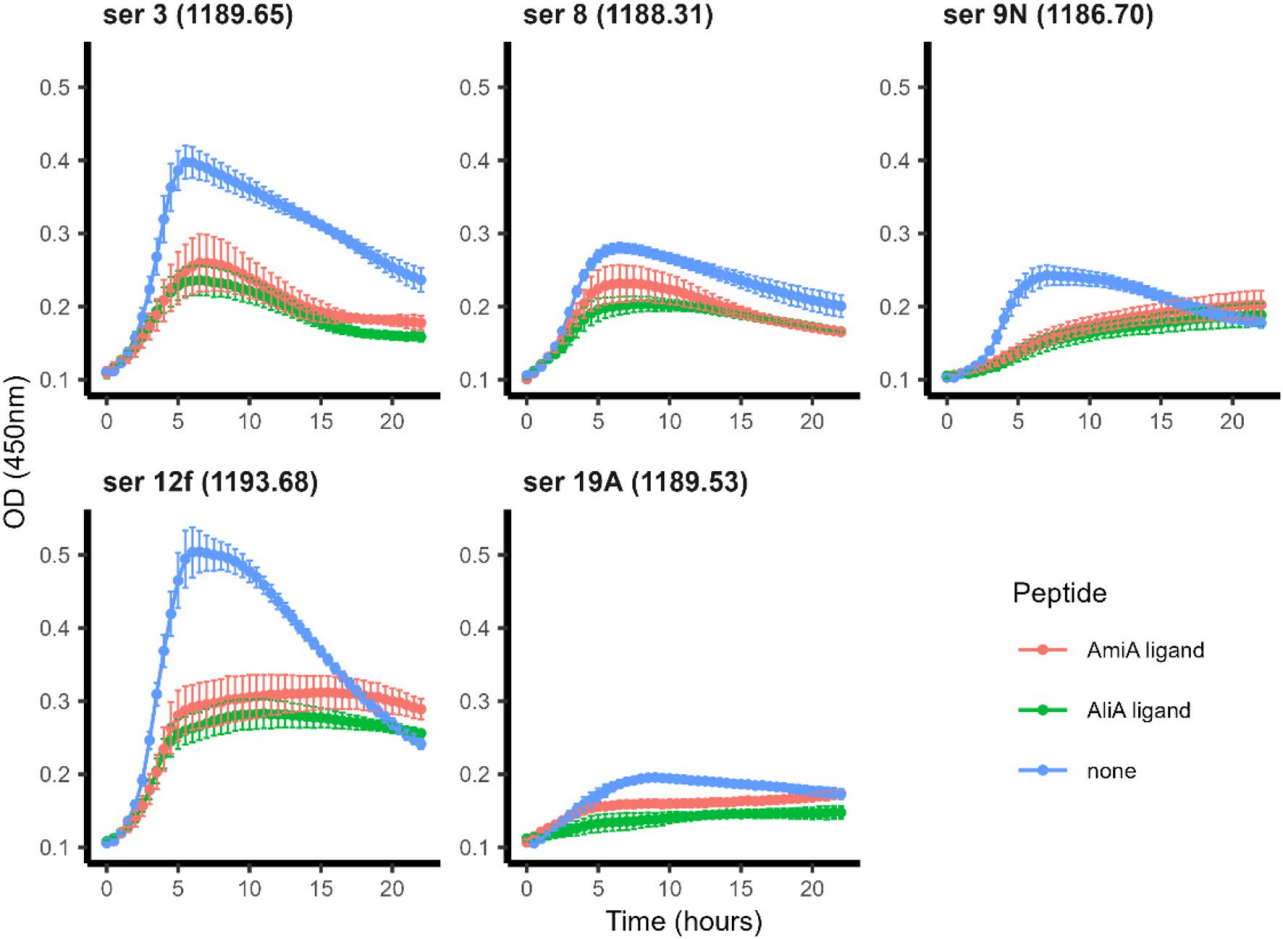
Growth curves of Swiss clinical isolates of *S. pneumoniae* in presence and absence of AmiA and AliA peptide ligands. Effect of peptides on growth was tested on isolates of different serotype (ser) with isolate number in brackets: 3 (1189.65), 8 (1188.31), 9N (1186.70), 12F (1193.68), 19A (1189.53). Growth curves were performed in peptide-free chemically defined medium (CDM) by measuring optical density OD (450 nm) over time in absence or presence of AmiA or AliA peptide ligands (0.5 mg/ml). Results represent 3 independent experiments, error bars indicate SEM.

We previously found that AmiA and AliA peptides inhibited growth of genetically diverse pneumococcal strains of serotypes 1, 2, 6B, 7F, 18C, 19A and non-typeable^7,8^. As the AmiA and AliA peptide ligands are of potential interest as therapeutics to treat pneumococcal diseases, we tested their effect on growth of Swiss clinical pneumococcal isolates from 2019 of the most prevalent serotypes causing invasive disease in Switzerland^9^: 3, 8, 9N, 12F and 19A. These serotypes were also prevalent in pneumococcal infections, invasive pneumococcal disease or amongst the most virulent serotypes in South Africa and China^10,11^. Both peptides reduced pneumococcal growth of all isolates tested (Fig. 7). In summary, the AmiA and AliA peptide ligands reduced growth of clinical pneumococcal isolates of serotypes 3, 8, 9N, 12F and 19A.

### No toxic effect of AmiA and AliA peptide ligands to human airway epithelial cells

Well-differentiated airway epithelial cell (AEC) cultures are organotypic cultures with air-liquid interface that mimic several aspects of the respiratory tract, including mucus production, airway cell heterogeneity and ciliary beating activity^12^. We evaluated whether the peptides are cytotoxic to primary human AEC (hAEC) cultures by incubating them with the peptides for 30 h, collecting medium from the apical and basolateral sides and determining the presence of LDH. LDH release was similar and low in media collected from hAEC cultures treated with or without peptides indicating no cytotoxicity (Supplementary Fig. S5a). Immunofluorescence staining showed the same cell morphology with ciliated cells and tight junctions present in all samples (Supplementary Fig. S5b). To summarize, we did not detect cytotoxic effects of the AmiA or AliA peptide ligands *in vitro* to hAEC cultures.

## Discussion

We found that the suppression of pneumococcal growth by the AmiA and AliA peptide ligands was dependent on uptake via the Ami permease, which altered the pneumococcal transcriptome. The peptides inhibited growth of pneumococcal isolates of currently-circulating serotypes not previously tested and were not cytotoxic to hAEC cultures.

We can associate growth inhibition and other previously observed phenotypic changes with alterations in the pneumococcal transcriptome caused by the AmiA or AliA peptide ligands. The genes most downregulated by both peptides were associated with energy or synthesis and transport of branched-chain amino acids (BCAAs).

BCAAs indicate the general nutritional status of bacterial cells and are an important constituent of the amino acid pool in bacterial proteins^13^. A feature of pneumococcal adaptation to nasopharyngeal colonisation is efficient biosynthesis, liberation or acquisition of BCAAs^14^. The BCAA transporter encoded by *liv* genes is necessary for disease pathogenesis as deletion led to reduced virulence *in vivo*^15^. The *ilv* operon encodes enzymes which condense threonine and pyruvate or two pyruvates into branched-chain keto acids, precursors of BCAAs^16^. Ablation of *ilvC* has been demonstrated to diminish BCAA concentration and impair growth, potentially by depriving the bacteria of nutrients in the stationary phase^13^. Both the AmiA and AliA peptide ligands downregulated *liv* and *ilv* genes. Designed antimicrobial peptide DM3 and penicillin also altered expression of genes in pneumococcal amino acid biosynthesis processes, most strongly affecting branched chain family amino acids^17^. Reduced ability to synthesise or acquire BCAAs might lead to the growth inhibiting effect of the AmiA and AliA peptides.

In *Staphylococcus aureus*, BCAA metabolism controls the phospholipid structure as BCAAs, mainly isoleucine, are converted to precursor molecules used to initiate fatty acid synthesis^18^. Altering the membrane fatty acid composition to modify membrane fluidity can be used in response to different environments. In *S. pneumoniae* fatty acid biosynthesis is regulated by the type II fatty acid biosynthesis cluster at a single genomic location and the transcriptional factor FabT^19^. The proteins FabK, FabG and FabF, which are encoded in the *fabKDGF-accB-fabZ-accCDA* operon, participate in enzymatic reactions of fatty acid biosynthesis by adding malonyl-coenzyme-A molecules to the growing hydrocarbon chain^20^. Alterations leading to changes in membrane fluidity have been associated with resistance mechanisms to intracellular oxidative stress^21^, importance for cell division site selection^22^ and FabT with indirect regulation of capsular polysaccharide^23^. It has been hypothesized that *S. pneumoniae* has the ability to alter membrane homeostasis in response to the production of quorum-sensing peptides, such as competence-associated peptide BriC which altered fatty acid biosynthesis^24^. It is interesting to note that penicillin treatment downregulated *fab* and *acc* genes^25^. AmiA and AliA peptide treatment upregulated *fab* and *acc* genes, which might alter fatty acid composition and be an adaptation mechanism of the pneumococcus to the peptide treatment in preparation for a changing environment. The above-mentioned indirect connections to cell division site selection and capsular polysaccharide may be relevant, as the peptides reduced pneumococcal growth and capsule size^8^. FtsE and FtsX are essential cell division proteins which interact with and control the peptidoglycan hydrolyse PcsB which separates daughter cells^26,27^. Their downregulation by the peptides might be a consequence of reduced growth which would require less expression of the daughter cell splitting machinery.

*S. pneumoniae* is naturally competent, which contributes to its genomic plasticity and can facilitate antigenic variation, vaccine escape and the acquisition of antibiotic resistance^28^. We cannot explain the reduction of transformation efficiency by AmiA and AliA peptide treatment with differential expression of *com* genes, which are involved in regulation and development of competence for genetic transformation^29^. Nevertheless, we found downregulation of other genes, namely *hrcA*, *grpE*, *dnaK* and *dnaJ*, which have been associated with regulating competence^30,31^. HrcA (heat-inducible transcription repressor), a key regulator of pneumococcal physiology, might repress the induction of competence in intracellular stress conditions and regulates *grpE* encoding a heat shock protein, *dnaK* and *dnaJ*, which both encode chaperone proteins^31^.

The* codY* gene, encoding a master nutritional regulator involved in amino acid metabolism^32^, was downregulated by both peptides. Among the genes regulated by CodY, we found downregulation of genes that encode the Ami permease, BCAA transporter (*liv* operon), *ilv* operon, *pcp*, *gdhA*, *asd*, *dapA* and *gapN*^32^. CodY has been hypothesized to sense peptide uptake through Ami and to be able to repress competence^33^. In *Streptococcus suis*, a mutation in *codY* decreased capsule thickness due to altered gene expression in sialic acid synthesis, leading to decreased sialic acid content in capsule^34^. Phenotypic changes related to CodY are interesting, as the AmiA and AliA peptide ligands decreased transformation efficiency and capsule size^8^.

In pneumococcus the translation inhibitors erythromycin, chloramphenicol, puromycin and tetracycline caused increased transcription of genes in purine biosynthesis, but antibiotics that target other cellular processes did not^35^. Furthermore, in response to these translation inhibitors, ribosomal proteins (*rpl* and *rps* genes) were upregulated and amino acid biosynthesis downregulated (including *ilvN*, *ilvC*, *ilvD*, *ilvE*, *trpA*, *trpC*, *trpD*, *trpG*). This reflects part of the observations of AmiA peptide ligand treatment, indicating that it might affect translation.

The effects of AmiA and AliA peptide ligands on the pneumococcal transcriptome had many similarities, but the regulation of genes involved in purine and pyrimidine biosynthesis and genes encoding ribosomal proteins was distinct for the AmiA peptide ligand. We hypothesize that the difference occurs intracellularly rather than depending on which substrate binding protein the peptides bind, since in D39 both peptides bind the AmiA protein^7^.

In conclusion, we found that the AmiA and AliA peptide ligands are taken up via the Ami-AliA/AliB permease into the pneumococcal cells. Together with our previous studies, we hypothesize that the peptides cause pneumococcal growth inhibition and phenotypic changes by modulating expression of genes involved in metabolism, as the processes affected were mostly associated with energy or synthesis and transport of amino acids. The peptides inhibited growth of clinical pneumococcal isolates of currently prevalent serotypes (3, 8, 9N, 12F and 19A) and we did not detect cytotoxic effects on human cells. AmiA and AliA peptide ligands may be useful tools to manipulate the transcriptome and control pneumococcal growth.

## Methods

### Bacterial strains and culture conditions

Bacterial strains, culture conditions and media were as previously^7^. Carlo Casanova kindly provided Swiss clinical *S. pneumoniae* strains. Daniel Straume kindly provided the D39 parent strain (ds865, D39 (∆*comAB::erm*)) and its mutant ∆*amiC* (ds1039, D39, ∆*amiC::Janus*). Peter W. M. Hermans kindly provided D39 parent strain and its D39 ∆*amiA* mutant, with construction of mutants described previously^5,36^*.*

### Peptides

Synthetic peptides (Genscript, Thermo Fisher Scientific) were ordered with ≥95 % purity. Peptides for fluorescent microscopy were labelled at the C-terminus with Lys (FITC).

### Growth assay

Growth assays were done as described previously^7^ and plotted in R, error bars indicate SEM of 3 independent biological replicates.

### Protein sequence alignment

Protein sequence alignment was done as described previously^7^.

### Epifluorescence microscopy

After overnight culture on agar plates, we grew *S. pneumoniae* D39 parent strains, D39∆*amiA* and D39∆*amiC* in BHI to OD_600nm_ = 0.1 to 0.2. Next, we centrifuged 1 ml culture for 1 min at 10,000 g and resuspended the pellet in 100 µl BHI and 50 µl of 1 mg/ml fluorescently labelled peptide. After incubating for 5 min, we washed the bacteria three times with 1 ml PBS and resuspended the pellet in 40 µl of PBS. Then, we spotted 1.5 µl onto an acrylamide pad inside a gene-frame on a microscope slide, which we prepared as described in the supplementary methods. We performed microscopy using a Zeiss Axio Imager M1 fluorescence microscope with a 100× oil immersion objective (EC Plan-Neufluoar 100x/1.30 Oil M27), filter with 493 nm excitation and 517 nm emission wavelength for the FITC channel and photographed images with a Zeiss Axiocam 712 mono camera. We edited pictures with the same settings for all samples to maintain comparability between samples and intensity of fluorescence using the Software Zeiss ZEN 3.8.: first, we applied denoising to the brightfield channel picture setting real wavelets to 1.0, then we adjusted the histogram upper white limit in the FITC channel to 5,000.

### Gene expression analysis by RNA-Seq

After overnight culture on agar plates, we grew *S. pneumoniae* strain D39 in BHI + 5 % FCS to OD_600nm_ = 0.5 and sub-cultured 100 µl of overnight culture in 10 ml of fresh CDM until OD_600nm_ = 0.2. We split the culture into two 5 ml cultures and added 0.5 mg/ml peptide to one sample. After 15 min incubation at 37 °C, we stopped transcription with RNAprotect bacteria reagent (Qiagen) and isolated total RNA with the RNeasy kit (Qiagen) following the manufacturer's instructions. We assessed RNA concentration and integrity with Nanodrop (Thermo Scientific) and Agilent Biolanalyzer (Agilent) and submitted 3 biological replicates of each sample to the Next Generation Sequencing Platform (University of Bern). There, quality control assessment, rRNA depletion, generation of libraries and sequencing were carried out as described in the Supplementary Methods.

Lexogen (Vienna, Austria) performed quality control, read processing and differential gene expression analysis up to the log fold change table as follows: quality control (fastqc), UMI extraction (umi_tools extract), trimming (cutadapt), STAR alignment to the reference genome (*S. pneumoniae* D39), umi deduplication (umi_tools dedup) and differential gene expression analysis with DESeq2.

For the heatmap in Fig. 4, we clustered differentially expressed genes with the k-means clustering algorithm using ComplexHeatmap^37^ in R. We submitted each generated cluster to DAVID 2021 (version of December 2021)^38^ to identify functional enrichment.

With the most differentially expressed genes based on their log2FC value, we retrieved a STRING network for *S. pneumoniae* D39 in Cytoscape 3.8.0 to create a gene association network.

### Gene expression analysis by RT-qPCR

We synthesised cDNA using SuperScript IV Reverse transcriptase (Invitrogen), including RNaseOUT Recombinant Ribonuclease Inhibitor (Invitrogen), according to the manufacturer's instructions. We performed RT-qPCR according to the manufacuturer's instructions using TaqMan Fast Advanced Master Mix (Applied Biosystems) and Custom TaqMan Gene Expression Assays (Applied Biosystems) with sequences in Supplementary Table S2.

### Human airway epithelial cell (hAEC) culture generation

We cultured hAEC cultures in 6.5 mm Transwells® on a 0.4 µm pore polyester membrane insert in flat bottom 24-well plates (Corning, 3470) as described previously^12^. We seeded 1x10^5^ cells per insert and performed experiments once a pseudostratified layer of differentiated airway epithelial cells was formed, including physiological mucus production and cilia movement.

### Cytotoxicity assay in hAEC cultures

We determined cytotoxicity of 30 h peptide incubation on hAEC cultures by measuring release of lactate dehydrogenase (LDH), a stable cytosolic enzyme released upon cell lysis, and visualizing the ultrastructure of the epithelium as done previously^12^. To the apical side of hAEC cultures we added 40 µl of TEER solution (NaCl 0.9 %, CaCl_2_ 1.25 mmol/l and HEPES 10 mmol/l dissolved in distilled water) as negative control, 40 µl of peptide resuspended in TEER solution (0.25 mg/ml, 0.5 mg/ml) and 40 µl of 1x LDH+ control solution from the CytoTox® Non-Radioactive Cytotoxicity Assay (Promega). After incubation for 30 h at 37 °C and 5 % CO_2_, we added 160 µl TEER to the apical side and collected the medium from the apical and basolateral side of each culture to determine cytotoxicity. We measured release of LDH on hAEC cultures using the CytoTox® Non-Radioactive Cytotoxicity Assay (Promega) according to the manufacturer's instructions and calculated %LDH release using the formula of Rayamajhi et al.^39^.

### Ethics

All research was performed in accordance with the relevant guidelines and regulations. For the primary human airway epithelial cell culture model the anonymized post-mortem tissue material was obtained through the Tissue Bank Bern (TBB) in accordance with ethical approval (KEK-BE 1571/2019).

### Supplementary Information

Supplementary Information.

## Data Availability

The transcriptomic data for this study has been deposited in the European Nucleotide Archive (ENA) at EMBL-EBI under accession number PRJEB74191.

## References

[1] Asokan GV, Ramadhan T, Ahmed E, Sanad H (2019). WHO Global Priority Pathogens List: A Bibliometric Analysis of Medline-PubMed for Knowledge Mobilization to Infection Prevention and Control Practices in Bahrain. Oman Med. J..

[2] van der Poll T, Opal SM (2009). Pathogenesis, treatment, and prevention of pneumococcal pneumonia. Lancet.

[3] Cillóniz C, Garcia-Vidal C, Ceccato A, Torres A, Fong I, Shlaes D, Drlica K (2018). Antimicrobial Resistance Among *Streptococcus pneumoniae*. Antimicrobial Resistance in the 21st Century.

[4] Claverys JP, Grossiord B, Alloing G (2000). Is the Ami-AliA/B oligopeptide permease of *Streptococcus pneumoniae* involved in sensing environmental conditions?. Res. Microbiol..

[5] Kerr AR (2004). The Ami-AliA/AliB permease of *Streptococcus pneumoniae* is involved in nasopharyngeal colonization but not in invasive disease. Infect. Immun..

[6] Nasher F, Heller M, Hathaway LJ (2018). *Streptococcus pneumoniae* proteins AmiA, AliA, and AliB bind peptides found in ribosomal proteins of other bacterial species. Front. Microbiol..

[7] Lux J (2022). AmiA and AliA peptide ligands are secreted by *Klebsiella pneumoniae* and inhibit growth of *Streptococcus pneumoniae*. Sci. Rep..

[8] Nasher F (2018). Peptide Ligands of AmiA, AliA, and AliB Proteins Determine Pneumococcal Phenotype. Front. Microbiol..

[9] Oyewole OR (2021). The Impact of Pneumococcal Conjugate Vaccine (PCV) Coverage Heterogeneities on the Changing Epidemiology of Invasive Pneumococcal Disease in Switzerland, 2005–2019. Microorganisms.

[10] Müller A (2022). *Streptococcus pneumoniae* Serotypes Associated with Death, South Africa, 2012–2018. Emerg. Infect. Dis..

[11] Zhou M (2021). Serotype Distribution, Antimicrobial Susceptibility, Multilocus Sequencing Type and Virulence of Invasive *Streptococcus pneumoniae* in China: A Six-Year Multicenter Study. Front. Microbiol..

[12] Gultom M, Laloli L, Dijkman R (2020). Well-differentiated primary mammalian airway epithelial cell cultures. Methods Mol. Biol..

[13] Kim GL (2017). Effect of decreased BCAA synthesis through disruption of ilvC gene on the virulence of *Streptococcus pneumoniae*. Arch. Pharm. Res..

[14] Green AE (2023). Airway metabolic profiling during *Streptococcus pneumoniae* infection identifies branched chain amino acids as signatures of upper airway colonisation. PLoS Pathog..

[15] Basavanna S (2009). Screening of *Streptococcus pneumoniae* ABC transporter mutants demonstrates that LivJHMGF, a branched-chain amino acid ABC transporter, is necessary for disease pathogenesis. Infect. Immun..

[16] Härtel T (2012). Characterization of central carbon metabolism of *Streptococcus pneumoniae* by isotopologue profiling. J. Biol. Chem..

[17] Le CF, Gudimella R, Razali R, Manikam R, Sekaran SD (2016). Transcriptome analysis of *Streptococcus pneumoniae* treated with the designed antimicrobial peptides, DM3. Sci. Rep..

[18] Frank MW, Whaley SG, Rock CO (2021). Branched-chain amino acid metabolism controls membrane phospholipid structure in *Staphylococcus aureus*. J. Biol. Chem..

[19] Lu YJ, Rock CO (2006). Transcriptional regulation of fatty acid biosynthesis in *Streptococcus pneumoniae*. Mol. Microbiol..

[20] Heath RJ, White SW, Rock CO (2001). Lipid biosynthesis as a target for antibacterial agents. Progress Lipid Res..

[21] Neville SL (2020). Cadmium stress dictates central carbon flux and alters membrane composition in *Streptococcus pneumoniae*. Commun. Biol..

[22] Mohedano ML (2005). Evidence that the essential response regulator YycF in *Streptococcus pneumoniae* modulates expression of fatty acid biosynthesis genes and alters membrane composition. J. Bacteriol..

[23] Zhang J (2021). Inactivation of Transcriptional Regulator FabT Influences Colony Phase Variation of *Streptococcus pneumoniae*. mBio.

[24] Aggarwal SD (2021). Competence-Associated Peptide BriC Alters Fatty Acid Biosynthesis in *Streptococcus pneumoniae*. mSphere.

[25] Rogers PD (2007). Gene expression profiling of the response of *Streptococcus pneumoniae* to penicillin. J. Antimicrob. Chemother..

[26] Alcorlo M, Straume D, Lutkenhaus J, Håvarstein LS, Hermoso JA (2020). Structural Characterization of the Essential Cell Division Protein FtsE and Its Interaction with FtsX in *Streptococcus pneumoniae*. Mbio.

[27] Bajaj R (2016). Biochemical characterization of essential cell division proteins FtsX and FtsE that mediate peptidoglycan hydrolysis by PcsB in *Streptococcus pneumoniae*. Microbiologyopen.

[28] Winkler ME, Morrison DA (2019). Competence beyond genes: Filling in the details of the pneumococcal competence transcriptome by a systems approach. J. Bacteriol..

[29] Piotrowski A, Luo P, Morrison DA (2009). Competence for genetic transformation in *Streptococcus pneumoniae*: termination of activity of the alternative sigma factor ComX is independent of proteolysis of ComX and ComW. J. Bacteriol..

[30] Slager J, Aprianto R, Veening J-W (2019). Refining the pneumococcal competence regulon by RNA sequencing. J. Bacteriol..

[31] Kwun MJ, Ion AV, Oggioni MR, Bentley SD, Croucher NJ (2023). Diverse regulatory pathways modulate bet hedging of competence induction in epigenetically-differentiated phase variants of *Streptococcus pneumoniae*. Nucl. Acids. Res..

[32] Hendriksen WT (2008). CodY of *Streptococcus pneumoniae*: link between nutritional gene regulation and colonization. J. Bacteriol..

[33] Caymaris S (2010). The global nutritional regulator CodY is an essential protein in the human pathogen *Streptococcus pneumoniae*. Mol. Microbiol..

[34] Feng L (2016). The CodY regulator is essential for virulence in *Streptococcus suis* serotype 2. Sci. Rep..

[35] Ng WL, Kazmierczak KM, Robertson GT, Gilmour R, Winkler ME (2003). Transcriptional regulation and signature patterns revealed by microarray analyses of *Streptococcus pneumoniae* R6 challenged with sublethal concentrations of translation inhibitors. J. Bacteriol..

[36] Alloing G, de Philip P, Claverys J-P (1994). Three Highly Homologous Membrane-bound Lipoproteins Participate in Oligopeptide Transport by the Ami System of the Gram-positive *Streptococcus pneumoniae*. J. Mol. Biol..

[37] Gu Z (2022). *Complex heatmap visualization*. Imeta.

[38] Sherman BT (2022). DAVID: A web server for functional enrichment analysis and functional annotation of gene lists (2021 update). Nucl. Acids Res..

[39] Rayamajhi M, Zhang Y, Miao EA (2013). Detection of pyroptosis by measuring released lactate dehydrogenase activity. Methods Mol. Biol..

