# Supplementary material for: AmiA and AliA peptide ligands, found in Klebsiella pneumoniae, are imported into pneumococci and alter the transcriptome
Source: Sci Rep. 2024 May 30;14:12416. doi: 10.1038/s41598-024-63217-2 (PMC11139975; doi:10.1038/s41598-024-63217-2)
Supplement: Supplementary file 1 — Supplementary Information. [file 41598_2024_63217_MOESM1_ESM.docx]

SUPPLEMENTARY INFORMATION

AmiA and AliA peptide ligands, found in Klebsiella pneumoniae, are imported into pneumococci and alter the transcriptome

This file includes:

Supplementary Methods

Supplementary Figures

Supplementary Tables

**SUPPLEMENTARY METHODS**

**Acrylamide slide preparation.** We prepared acrylamide slides by sticking a gene-frame (Thermo Scientific, AB0578) onto a glass slide and mixing the following reagents in a tube per gene frame: 370 µl PBS, 125 µl 40 % acrylamide/bis-acrylamide 29:1 (Sigma-Aldrich, A7802), 0.5 µl TEMED (Sigma-Aldrich, T9281), 5 µl freshly prepared 10 % APS (Sigma-Aldrich, A3678). From this mix we poured 500 µl into the gene-frame and added another glass slide to close it. After 30 min incubation at room temperature, we gently slid off one glass slide, cut the acrylamide pads into small pieces and incubated them in PBS for at least 30 min before use.

**Gene expression analysis by RNA-Seq.** At the next Generation Sequencing Platform (University of Bern), the quantity and quality of purified total RNA was assessed using the Qubit 4.0 fluorometer (Thermo Fisher Scientific) with the Qubit RNA BR Assay Kit (Thermo Fisher Scientific, Q10211) and an Advanced Analytical Fragment Analyzer System using a Fragment Analyzer RNA Kit (Agilent, DNF-471). After, 150 ng of input RNA were depleted of ribosomal RNA with a RiboCop rRNA Depletion Kit for Gram Positive Bacteria (G+) (Lexogen, SKU 127) following the Lexogen User guide 125UG246V0102. Next, cDNA libraries were made with the CORALL RNA-Seq V2 Library Prep Kit with UDI 12 nt Set A3 (Lexogen, SKU 173) according to the RTL protocol with 15 PCR cycles (Lexogen User guide 171UG394V0100). The resulting cDNA libraries were evaluated using the Qubit 4.0 fluorometer with the Qubit dsDNA HS Assay Kit (Thermo Fisher Scientific, Q32854) and an Agilent Fragment Analyzer with a HS NGS Fragment Kit (Agilent, DNF-474). Pooled cDNA libraries were sequenced paired-end using NextSeq 1000/2000 P2 Reagents v3 (200 cycles; Illumina, 20046812) on an Illumina NextSeq 1000 instrument. On average the run produced 26 million reads/library. The quality of the sequencing run was assessed with the Illumina Sequencing Analysis Viewer (version 2.4.7) and all base call files were demultiplexed and converted into FASTQ files with Illumina bcl2fastq conversion software (v2.20).


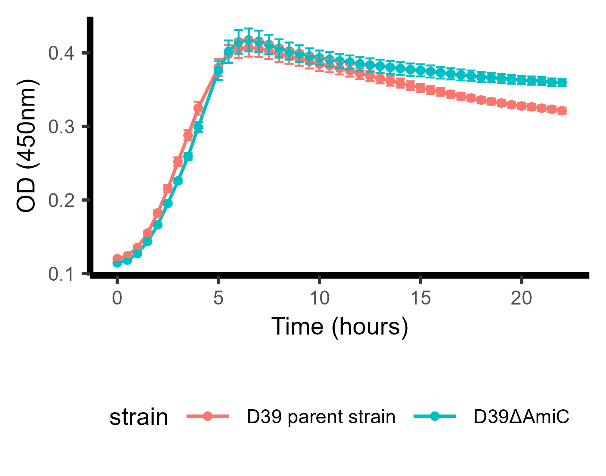
**SUPPLEMENTARY FIGURES**

**Supplementary Figure S1.** Growth curves of *S. pneumoniae* D39 parental strain and D39 ∆*amiC* mutant*.* Growth curves were performed in peptide-free chemically defined medium (CDM) by measuring optical density OD (450 nm) over time. Results represent 3 independent experiments, error bars indicate SEM.

| **a** | D39 parental strain | D39 ∆*amiA* |
| --- | --- | --- |
| FITC | 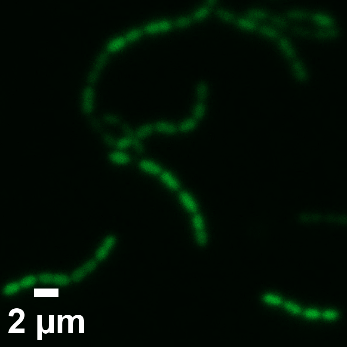 | 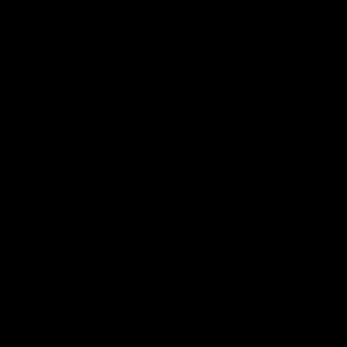 |
| BF | 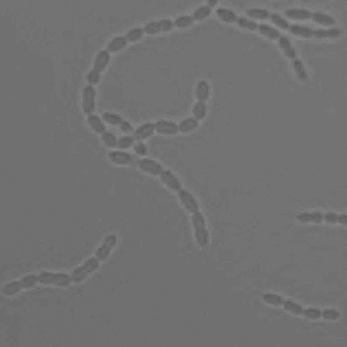 | 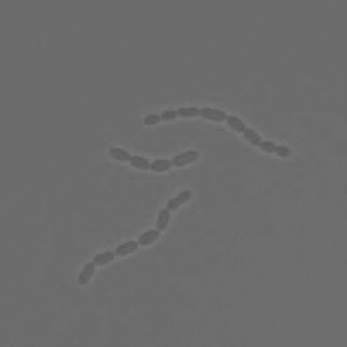 |
| **b** |  |  |
| FITC | 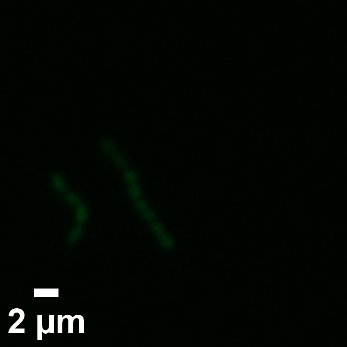 | 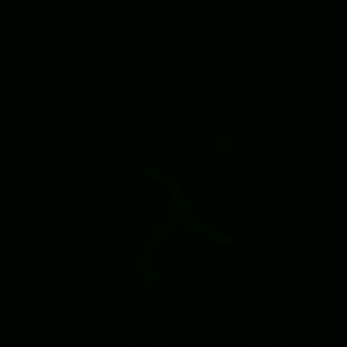 |
| BF | 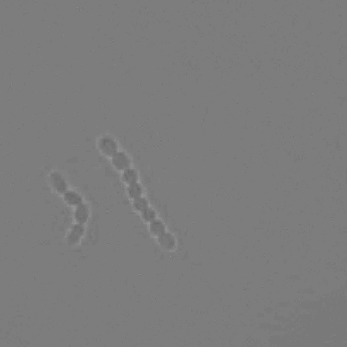 | 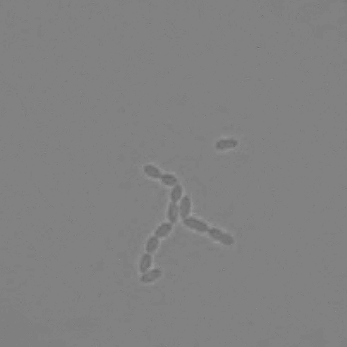 |

**Supplementary Figure S2.** Epifluorescence microscopy pictures of pneumococcal cells of *S. pneumoniae* D39 parental strain and D39 ∆*amiA* after incubation with FITC-labelled AmiA peptide ligand **(a)** and AliA peptide ligand **(b)** taken at mid-bacterium localization in z position showing FITC and Brightfield (BF) channel. Scale bar indicates 2 µm for all pictures in a and b.


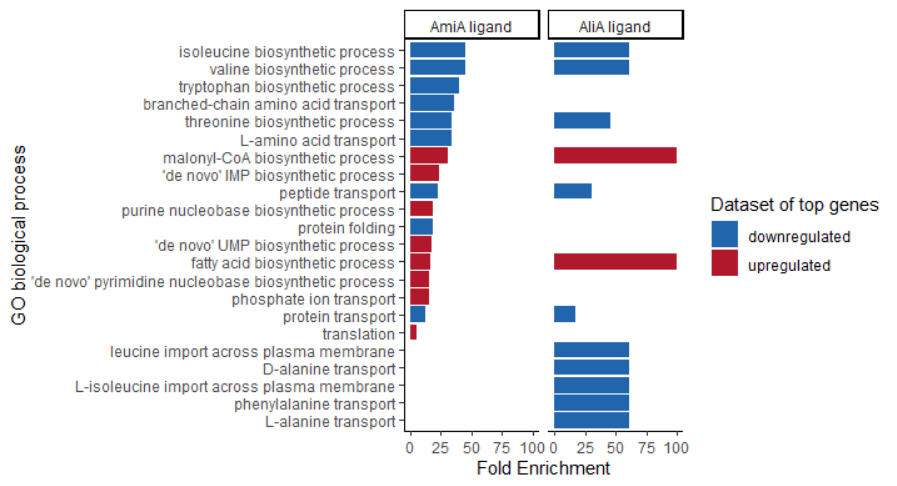
**Supplementary Figure S3.** Gene Ontology (GO) biological process enrichment in top up-, or downregulated genes after peptide treatment of *S. pneumoniae* strain D39. Enrichment was found in dataset of significantly (p value < 0.05) downregulated genes (log2FC ≤ -1.25) with bars in blue and upregulated genes (log2FC ≥ 1.25) with bars in red compared to the genome background in *S. pneumoniae* D39 after treatment with AmiA or AliA peptide ligands.


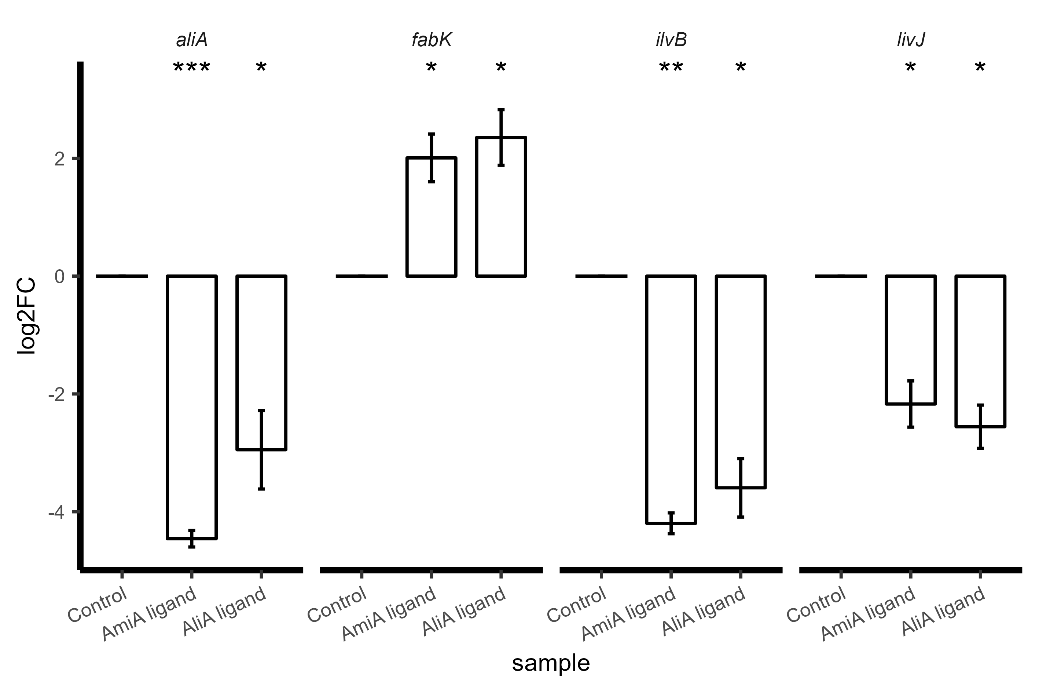
**Supplementary Figure S4.** Confirmation of differential gene expression after AmiA or AliA ligand treatment of *S. pneumoniae* D39 by real-time RT-PCR. The data represents 3 biological replicates. * indicates p value ≤ 0.05, **: p value ≤ 0.01, ***: p value ≤ 0.001 by pairwise t-test. For AmiA and AliA peptide ligand treatment respectively p values are 0.00099 and 0.04753 for *aliA*, 0.038 and 0.038 for *fabK*, 0.0018 and 0.0186 for *ilvB*, 0.031 and 0.020 for *livJ*.

| **a** | 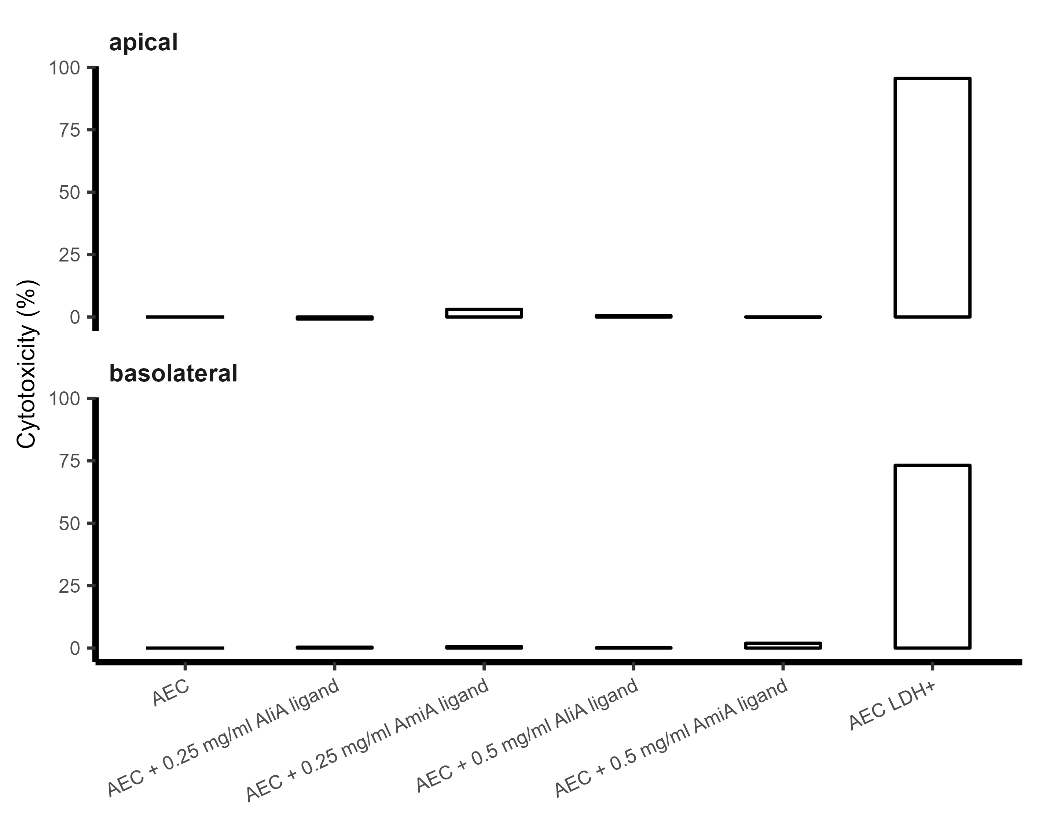 | | | |
| --- | --- | --- | --- | --- |
| **b** | No peptide | AmiA ligand | AliA ligand |  |
|  | 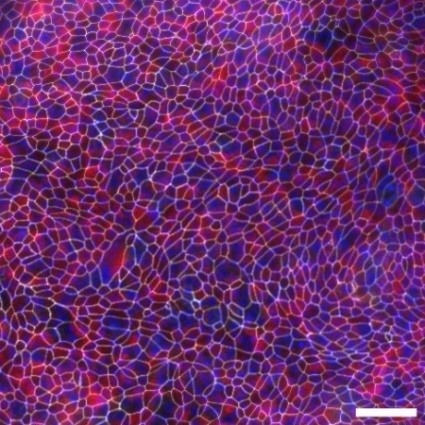 | 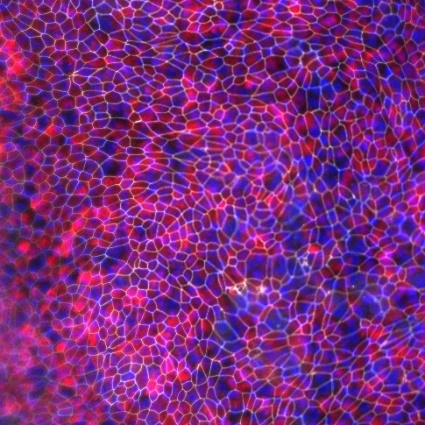 | 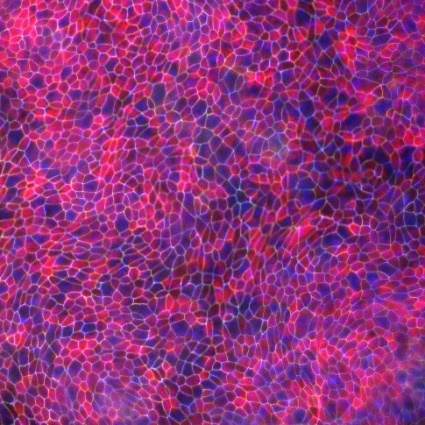 | 0.25 mg/ml peptide |
|  | 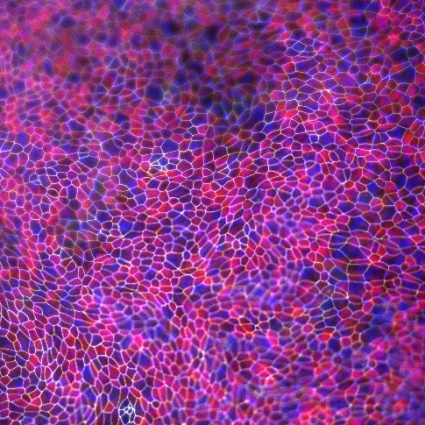 | 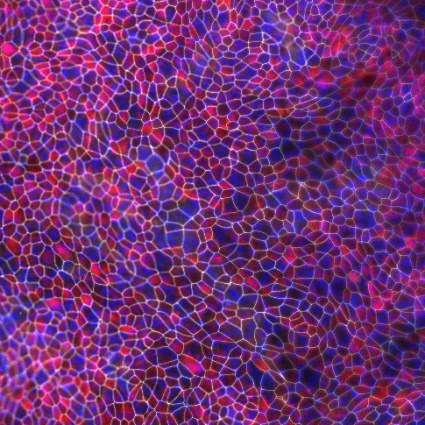 | 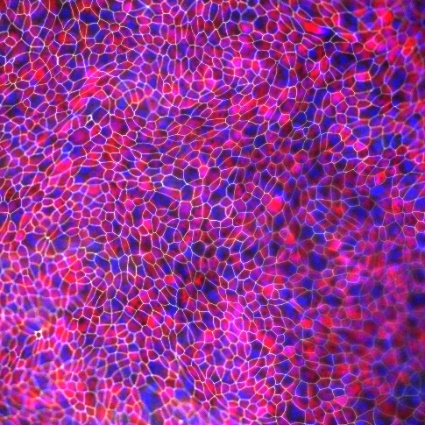 | 0.5 mg/ml peptide |

**Supplementary Figure S5.** No toxic effect was detected for AmiA or AliA peptide ligands to human airway epithelial cell cultures. **(a)** Release of lactate dehydrogenase (LDH) was low in hAEC (human airway epithelial cell) cultures (apical and basolateral side) untreated or treated with AmiA and AliA peptide ligands for 30 h, indicating no cytotoxicity. LDH+ control was used to assess 100 % cytotoxicity. **(b)** Immunofluorescence staining of hAEC cultures with or without peptides. AEC cultures without peptide have the same morphology as AEC cultures treated for 30 h with AmiA or AliA peptide ligands (0.25 mg/ml or 0.5 mg/ml). The images were taken with 40x objective, blue: DAPI for nucleus, red: β-Tubulin IV for ciliated cells, grey: ZO-1 for tight junctions. Scale bar indicates 50 µm for all images.

**SUPPLEMENTARY TABLES**

| **Gene** | **SPD locus** | **Log2FC AmiA ligand** | **Log2FC AliA ligand** |
| --- | --- | --- | --- |
| ***asd*** | SPD_0900 | -2.7 | -3.1 |
| ***codY*** | SPD_1412 | -1.5 | -1.4 |
| ***dapA*** | SPD_0901 | -2.4 | -2.7 |
| ***dnaJ*** | SPD_0461 | -2.1 | -1.3 |
| ***dnaK*** | SPD_0460 | -2.0 | -1.1 |
| ***ftsE*** | SPD_0659 | -1.0 | -1.2 |
| ***ftsX*** | SPD_0660 | -1.2 | -1.3 |
| ***gapN*** | SPD_1004 | -4.3 | -3.2 |
| ***gdhA*** | SPD_1158 | -3.2 | -2.8 |
| ***hrcA*** | SPD_0458 | -1.9 | -1.3 |
| ***grpE*** | SPD_0459 | -1.9 | -1.2 |
| ***nanA*** | SPD_1504 | -1.4 | -0.4 |
| ***pcp*** | SPD_0753 | -3.0 | -2.7 |
| ***pcsB*** | SPD_2043 | -0.5 | -1.1 |

**Supplementary Table S1.** Differential gene expression in log2FC values caused by AmiA and AliA peptide ligands treatment on *S. pneumoniae* strain D39. All shown gene expression values were significant.

| **Forward Primer Sequence** | **Reverse Primer Sequence** | **Reporter 1 Dye** | **Reporter 1 Sequence** | **Reporter 1 Quencher** | **Assay Name** |
| --- | --- | --- | --- | --- | --- |
| TCCTATAAATACACATCTAAGACCAGCGA | CAACTGAGAGGCATAGGCTGTAC | FAM | CCTGACGGAAATCCTT | NFQ | ALIA |
| GGTAGCCACAGCTGTATCTATTCCT | GCCGCAGCACCTTCAC | FAM | CATCCGCAATTCCTCC | NFQ | FABK |
| CGAGTCATACAAGGGTGAAATCGTT | ACGATAGCATCAAAGTCTTTCCCTTTC | FAM | CCTGCTACGAAAGTTT | NFQ | LIVJ |
| TTGGAATGGGAGGCATGCA | TCATAAAGTCCGCTTCCGTCATG | FAM | TCGCAGCAAATATTG | NFQ | ILVB |
| GACGATACATAGCCGACCTGAGA | GTAGGAGTCTGGGCCGTGTCT | FAM | CCAGTGTGGCCGATC | NFQ | 16S |

**Supplementary Table S2.** Details for Custom TaqMan Gene Expression Assays.
